# Reduction of coherent artefacts in super‐resolution fluorescence localisation microscopy

**DOI:** 10.1111/jmi.12453

**Published:** 2016-08-19

**Authors:** PANTELIS GEORGIADES, VIKI J. ALLAN, MARK DICKINSON, THOMAS A. WAIGH

**Affiliations:** ^1^ Biological Physics, School of Physics and AstronomyThe University of ManchesterManchesterU.K.; ^2^ Faculty of Life Sciences, Michael Smith BuildingThe University of ManchesterOxford Road, ManchesterM13 9PTU.K.; ^3^ Photon Science InstituteThe University of ManchesterOxford Road, ManchesterM13 9PLU.K.

**Keywords:** Fluorescence, PALM, speckle, STORM, super‐resolution, TIRF

## Abstract

Super‐resolution localisation microscopy techniques depend on uniform illumination across the field of view, otherwise the resolution is degraded, resulting in imaging artefacts such as fringes. Lasers are currently the light source of choice for switching fluorophores in PALM/STORM methods due to their high power and narrow bandwidth. However, the high coherence of these sources often creates interference phenomena in the microscopes, with associated fringes/speckle artefacts in the images. We quantitatively demonstrate the use of a polymer membrane speckle scrambler to reduce the effect of the coherence phenomena. The effects of speckle in the illumination plane, at the camera and after software localisation of the fluorophores, were characterised. Speckle phenomena degrade the resolution of the microscope at large length scales in reconstructed images, effects that were suppressed by the speckle scrambler, but the small length scale resolution is unchanged at ∼30 nm.

## Lay description

Super‐resolution fluorescence localisation microscopy allows 20–30 nm resolution images to be created on fluorescently labelled specimens, which include intact biological cells. The diffraction limit of conventional optical microscopes is broken by building up the images point by point, localizing single fluorescent molecules in the image one by one. The majority of the fluorophores in the image are switched off (shelved in a dark state), whilst a small subset of them stochastically switch to a short lived fluorescent state so that their emission does not overlap. Such techniques have been very successful and led to the award of the Nobel Prize for chemistry in 2014. However, the lasers used to illuminate the specimen in such techniques can create artefacts in the images due to their high coherence. Light waves reflected from different parts of the microscope's optical components interfere with one another and create two closely related artefacts that reduce the quality of the images; fringes and speckle. Fringes are observed as stripes in the images (often with some curvature) and speckle patterns consist of a series of random blobs. Coherent light that is in phase causes the bright regions in the images (constructive interference), whereas out of phase light causes the dark regions (destructive interference). A large number of methods have been implemented in the literature with laser illuminated microscopes to remove such image artefacts. These include scanning the laser around the sample, launching the laser through a vibrated optical fibre and switching the laser on and off at high frequencies. We describe a new method to reduce coherent artefacts in optical microscopes, using a vibrating polymer membrane called a speckle scrambler. The speckle scrambler is demonstrated with super‐resolution fluorescence localisation microscopy. It performs well with this microscopy technique providing substantial improvements in resolution on intermediate length scales (μm), whereas the resolution at small length scales is unchanged (∼30 nm). The speckle scrambler is relatively easy to integrate in to the optical set up and compares favourably with alternative methods. Some of the measurements were performed with a total internal reflection fluorescence lens (TIRF). Such lenses are known to be particularly prone to coherence artefacts. The speckle scrambler also performed well with such TIRF lenses and thus appears also to be well suited to specialized single molecule experiments that use this type of apparatus.

## Introduction

The microscope, since its inception during the 16th century, has been a valuable tool in the hands of researchers, who have now gained insights in previously unresolved length scales down to the order of Angstroms (Henriques *et al*., 2011; Kourkoutis *et al*., 2012). The development of fluorescence microscopy, in which an image is formed from the detection of light from structures specifically tagged with fluorescent probes at molecular level resolution, has been of great importance in biology (Garini *et al*., 2005). The resolving power of any microscope is fundamentally dependent on the type of radiation used and results from the diffraction of light going through its optical elements. This physical barrier was first postulated by Abbe in 1873 and is approximately equal to half the wavelength of light used in the *x*–*y* plane of two dimensional images (Abbe, 1873). For visible light, the Abbe limit roughly translates to 250 nm in the lateral direction and 500 nm in the axial direction; a significant improvement over the capabilities of the naked eye, but nevertheless insufficient for ultra‐structural imaging in subcellular assemblies.

Several methods have recently been developed to overcome the diffraction limit and are referred to as super‐resolution fluorescence microscopy. One of these approaches relies on spreading the fluorescence signal in the temporal dimension, to the point where the signal from individual fluorophores is no longer overlapping. The subsequent localisation of the centroid of each emitting fluorophore allows for the reconstruction of a super‐resolved image. Examples of such single‐molecule localisation techniques are photoactivation localisation microscopy (PALM) and stochastic optical reconstruction microscopy (STORM) (Betzig *et al*., 2006; Rust *et al*., 2006; Davis, 2009), which are two conceptually similar methods of super‐resolution microscopy. In both techniques, lasers are the illumination method of choice, since they provide well collimated, narrow bandwidth light beams. The quasi‐monochromatic nature of laser radiation allows for it to be easily removed from the detection plane using the appropriate set of excitation and emission filters, allowing for high signal‐to‐noise ratio in the resulting fluorescent images (Heilemann, 2010). Furthermore, the wavelength of the lasers can be chosen to specifically match the fluorophores used, maximising the excitation efficiency, and they are well adapted to total internal reflection fluorescence microscopy (TIRFM) with many advantages over the traditional arc lamps previously used (Mattheyses *et al*., 2010). TIRF is often the method of choice for single molecule localisation experiments, since it provides a significant boost in signal‐to‐noise ratio, and thus lower localisation uncertainty in the lateral and axial positions of the fluorophores. (Novotny & Hecht, 2012).

However, using lasers to illuminate the sample plane is not always advantageous compared to arc lamps and LED light sources. The high spatial and temporal coherence of the laser light can give rise to undesirable interference patterns in the sample plane, as a result of scattering and partial reflection of the light from the microscope's optical elements (Goodman, 2010). These interference patterns can adversely affect the quality of the image both in TIRF and wide‐field imaging, since the uneven illumination makes it difficult to distinguish intensities in different spatial regions of the sample and thus makes quantitative analysis challenging (Johnson *et al*., 2012). Additionally, inherent imperfections and dust particles on the optical components’ surfaces can give rise to speckle, which manifests itself as a grainy pattern of spots on the illumination plane. Speckle is responsible for both increased noise and the reduction of resolution in laser‐based fluorescence microscopes (Goodman, 1976; Francon, 1977). In order to minimise speckle and interference in the imaging plane, a number of different approaches have been implemented in the past, such as the use of rotating diffusers/oscillating mirrors to destroy the temporal and spatial coherence of the laser beam, scanning of the beam across the samples, the passage of the beam through a vibrating glass fibre and high‐frequency oscillatory switching of solid state lasers to broaden the emission spectra (van't Hoff *et al*., 2008; Mattheyses *et al*., 2010; Johnson *et al*., 2012; Bosch *et al*., 2014; Dulin *et al*., 2014). Many publications to date in the super‐resolution imaging literature demonstrate speckle artefacts on their images, and it's often difficult to control interference and speckle phenomena in traditional designs for super‐resolution localisation microscopy (Bates *et al*., 2013; Betzig *et al*., 2006). Speckle patterns can evolve with time due to thermal drift, which is also a problem, since artefacts can quickly become worse as the apparatus becomes more misaligned after partial correction, providing significant background noise in software reconstruction algorithms (PALM/STORM).

Here, we use a speckle scrambler that incorporates an electroactive polymer to translate a diffuser perpendicular to the laser beam in order to eliminate both interference and speckle phenomena by scrambling the wavefront's phase and, thus, homogenising the light intensity across the field of view (Giger *et al*., 2012). We demonstrate the utility of the speckle scrambler using dip coated dextran films and lung fibroblast cells. Both systems were labelled with the same fluorophore, Alexa Fluor 647. The speckle scrambler provides a substantial improvement of the fidelity of super‐resolution images at intermediate length scales (μm), but the resolution is unchanged at small length scales.

## Materials and methods

### Super‐resolution microscope setup

**Figure 1:**
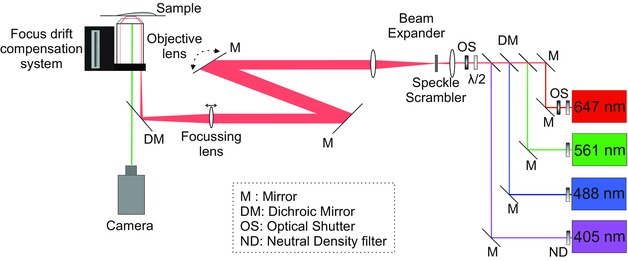
A schematic diagram of the optical arrangement of a PALM/STORM apparatus build around an Olympus IX‐71 inverted fluorescence microscope. The lasers are combined into a single beam using the appropriate dichroic mirrors (DM), passed through a half wave plate (λ/2) and focussed on the back focal plane of the TIRF objective lens through the back illumination port of the microscope. The sample is placed on a fully motorised *x*–*y* stage and the objective position is held constant relative to the sample using a focus drift compensation system. The fluorescent emission from the sample is detected using either an EMCCD or a sCMOS camera. The speckle scrambler is placed on the intermediate focal plane of a Keplerian beam expander.

A schematic diagram of the PALM/STORM microscope is shown in Figure 1. An Olympus IX‐71 inverted fluorescence microscope was used as a base for the system, equipped with the Olympus APON 60XOTIRFM (NA 1.42) and the Olympus UPAON 100XOTIRFM (NA 1.49) immersion oil, TIRF objective lenses. A fully motorised *x*–*y* stage (PRIOR HLD117) was controlled through a PRIOR ProScan III controller and a Mad‐City Labs C‐focus system was used to compensate for thermal drift in the z‐plane. Illumination was provided by Coherent (American company, California) OBIS 405 LX 200 mW, OBIS 488 LX 150 mW, Sapphire 561LP 50 mW and OBIS 647LX 120 mW lasers. Thorlabs (US based, New Jersey) lenses (LA series) with antireflecting coating in the visible spectrum and mirrors (BB05‐E01) were used to guide, expand and focus the laser beam onto the back focal plane of the objective. A Chroma TRF89902 Quad Band filter cube was used in the microscope to separate between the excitation and emission wavelengths. Finally, a Photometrics Evolve 512 EMMCCD and a Hamamatsu ORCA Flash v2 sCMOS camera were available for imaging, although the EMMCCD was predominantly used in the following experiments. The PALM/STORM microscopy is similar in design to that of Bates *et al*. (2013), with the inclusion of additional mirrors next to the lasers to facilitate alignment (to insure all four beams are collinear), additional mirrors before the microscope to facilitate switching between TIRF, SLIM field and far field imaging modalities and a beam expander with the speckle scrambler.

The speckle scrambler used was an Optotune (Swiss company) LSR‐3005‐17S‐VIS and was placed in the intermediate focal plane of the Keplerian beam expander as per the manufacturer's instructions. No static diffuser is included in this setup, since the beam needs to be collimated and focused after the beam expander, which would become very difficult with a static diffuser with a resultant highly divergent beam. The speckle scrambler consists of a diffuser bonded on a polymer membrane that includes four independent dielectric elastomer actuators (DEAs). Upon activation, the surface of the electrodes increases in size and causes a motion of the rigid diffuser in the membrane plane, thus homogenising the laser profile. The unit has a 5 mm aperture, operates at 300 Hz and the diffusion angle (FWHM) is 17º (Giger *et al*., 2012).

### Dextran STORM imaging

Dextran conjugated to Alexa Fluor 647 was purchased from Life Technologies (10 000 MW, anionic, fixable, catalog no: D‐22914) and was reconstituted according to the manufacturer's instructions. Thirty‐five millimetre MatTek glass bottom dishes (No 1.5 coverslip, thickness 0.170 ± 0.005 mm) were incubated for 10 min with poly‐l‐lysine solution (Sigma‐Aldrich P4707) at room temperature, followed by a 10 min incubation with a 20 ng mL^–1^ solution of Dextran/A647 and three 5 min washes with double distilled H_2_O. This allowed for the production of uniform and reproducible dextran films. Fresh samples were made for each experiment (Metcalf *et al*., 2013).

The imaging buffer used during data acquisition consisted of 50 mM Tris, 10 mM NaCl, 10 % w/v glucose, 10 mM Cysteamine (MEA), 0.3 mg mL^–1^ glucose oxidase, 40 μg mL^–1^ catalase and the pH was adjusted to 8 (Dempsey *et al*., 2011). All the chemicals used were purchased from Sigma‐Aldrich. During imaging, Sigma‐Aldrich 56822 immersion oil was used.

Data was acquired using the EMCCD camera using an integration time of 10 ms, set at 6 e^−^/ADU, with an offset bias of 500 ADU and linear gain factor of 100. A 60× TIRF (NA 1.42) lens was used during imaging, so that a relatively large field of view was accessible. A total of 10 000 frames were recorded for each dataset, using a window of 256 × 256 pixels in the centre of the camera's sensor. The data acquisition was performed using the Micro‐Manager suite and the super‐resolution data was reconstructed using the ImageJ plugin ThunderSTORM (Edelstein *et al*., 2010; Ovesný *et al*., 2014). Images were collected with and without the speckle scrambler in the Keplerian beam expander. The fitting constraints implemented in fits of the STORM data in ThunderSTORM were contained in the Wavelet (B‐Spline) image filter (the B‐spline order was 3 and the B‐spline scale was 2). Approximate localisation of the fluorescent molecules was achieved using the local maximum method (1.5 times the standard deviation of the signal‐to‐noise ratio threshold applied, with 8‐neighbourhood connectivity). Finally, the subpixel localisation was performed by fitting the point spread function with an integrated Gaussian function with a 3 pixel fitting radius and the fit was optimised using the weighted least squares method.

### MRC5 cell imaging

MRC5 lung fibroblast cells were cultured in 75 cm^2^ Corning T‐75 flasks, in Dulbecco's Modified Eagle's Medium/Nutrient Mixture F‐12 Ham supplemented with 10 % w/v fetal bovine serum, penicillin and streptomycin. Prior to imaging, cells were placed onto 35 mm MatTek glass bottom dishes (No 1.5 coverslip, 0.170±0.005 mm) until the desired confluency was achieved. The MRC5 cells were fixed with ‘fix‐perm’ solution at 37°C (3.7 % w/w formaldehyde, 0.1 % w/w glutaraldehyde and 1% w/w Triton X‐100 in buffer A (80 mM K‐PIPES,1 mM MgCl_2_, 5 mM EGTA, 4 % w/w PEG‐8000 at pH 6.8)) for 5 min, then incubated for a further 15 min in fixative solution (3.7 % w/w formaldehyde and 0.075 % w/w glutaraldehyde in Buffer A). Finally, the cells were incubated for 30 min in a 0.1 % w/w Triton X‐100 solution in Buffer A to permeabilise the membrane barriers and the reaction was quenched by washing 3 times with a 0.1 % w/w NaBH_4_ solution in PBS. The cells were subsequently stained using a DM1A mouse anti‐α‐tubulin antibody (dilution 1:500) and Alexa Fluor 647 donkey anti‐mouse secondary (dilution 1:500) antibodies (purchased from Jackson ImmunoResearch). Microtubular filaments within the cells were thus fluorescently labelled using this procedure.

First, wide‐field, diffraction limited images were recorded, using minimal illumination intensity with the 647 nm laser to avoid photobleaching, with the speckle scrambler in both the ON and OFF states. Subsequently, the STORM data was acquired by increasing the illumination intensity and controlling the fraction of the fluorophores in the fluorescent state using the 405 nm laser's intensity. A total of 25 000 frames were recorded using the 100× TIRF (NA 1.49) objective lens for each super‐resolved image. Finally, the STORM data were processed with ThunderSTORM, using similar constraints as with the Dextran film STORM data. The subpixel localisation fitting radius was adjusted to 5 pixels due to the slightly higher magnification objective lens (100× vs. 60×). In order to evaluate the illumination uniformity gain with the speckle scrambler, sequential imaging of the same FOV was performed with the speckle scrambler ON and OFF.

### Beam profile

The laser beam profile was imaged using a Thorlabs BC‐106N‐VIS CCD camera beam profiler, which was placed just above the objective lens. This allowed us to directly image the collimated beam (focused on the back focal plane of the 100× objective) without a sample on the objective.

## Results

**Figure 2:**
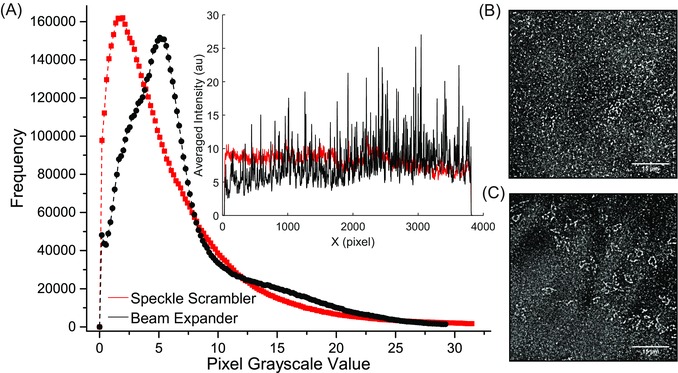
(A) Comparison of the distribution of pixel greyscale values of the super‐resolution reconstructed images from a Dextran/A647 coated glass bottom dish when imaged with (B) and without (C) the use of the speckle scrambler. The illumination of the sample is noticeably more uniform on the images after the introduction of the speckle scrambler (red) compared to the one without it (black). Additionally, a comparison of the intensity profiles along the *x*‐axis of both images is shown in the inset of (A). The intensity along the *y*‐axis was averaged and plotted as a function of the *x*‐coordinate for both images. There is significantly less variation in minima and maxima after the introduction of the speckle scrambler.

Figure 2 shows the intensity histograms of super‐resolved, reconstructed images collected for a Dextran/A647 coated glass bottom dish with (B) and without (C) the speckle scrambler in the beam expander optics (data collected in TIRF mode). Furthermore, a line profile along the *x*‐axis in both images is shown in the inset of Figure 2(A). The fluorescently labelled dextran film was used to provide a uniform and reproducible calibrant for the fluorescence microscopy experiments. When using the speckle scrambler, the histogram of the pixels’ greyscale value of the reconstructed image is a smoother Gaussian profile, whereas without it, the greyscale distribution has an exaggerated tail towards both ends of the spectrum, as a result of uneven illumination. Similarly, the line profile of the image obtained using the speckle scrambler displays significantly less variation in intensity when compared to the image without it.

**Figure 3:**
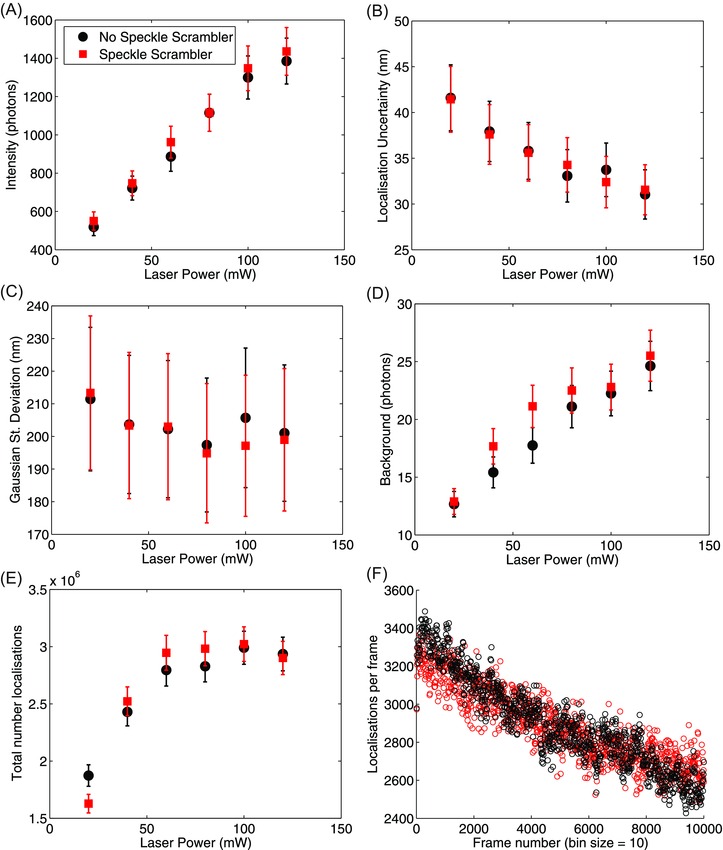
The fit parameters obtained from the statistical analysis of STORM data using ThunderSTORM (Ovesný *et al*., 2014) on Dextran/A647 data obtained with the speckle scrambler (red) and without it (black). (A) The intensity per localisation event for both setups indicates identical behaviour of the fluorophores, and varying the laser power again yields similar results within error. Additionally, the localisation uncertainty (B), the Gaussian fit standard deviation (C), the background noise in photons (D) and the total number of localisations (E) show similar behaviour (within 1 SD; 95%CI) with respect to laser power. (F) The localisations per frame acquired using 100 mW of laser power with and without the speckle scramble. The data shown is the mean value for each parameter obtained from three independent measurements and the error bars represent the standard deviation (A)–(E).

Results from fits obtained using ThunderSTORM to analyse the data are shown in Figure 3 (Ovesný *et al*., 2014). The experiment was repeated three times for each setup, using a range of laser powers, up to 120 mW, which is the maximum power of our 647 nm laser line, both with and without the speckle scrambler. The intensity per localisation (the number of photons collected for each localisation event, Fig. 3A), localisation uncertainty (∼30 nm at high laser powers, Fig. 3B), the Gaussian fit standard deviation (fitted point spread function FWHM/2.3, Fig. 3C), the background number of photons (Fig. 3D) and the number of localisations (Figs. 3E and F) remain unchanged within 1 standard deviation (95% CI) between setups.

**Figure 4:**
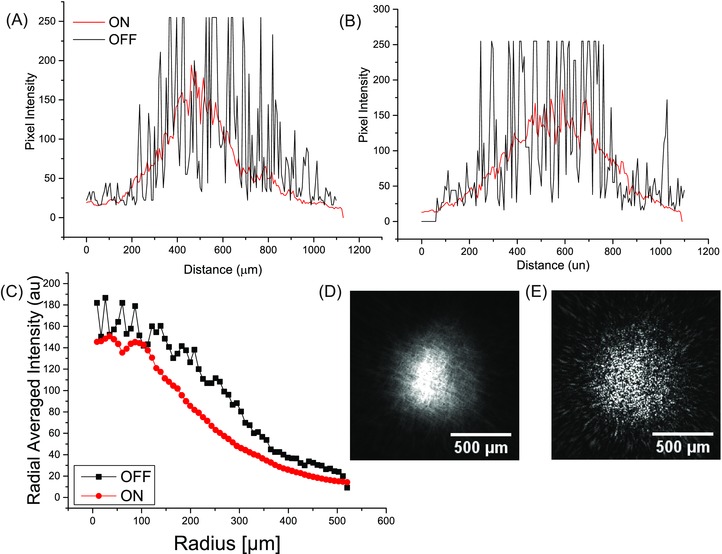
The intensity profile of the CCD array across the *x*‐axis (A) and *y*‐axis (B). The intensity cross‐sections with the speckle scrambler in the OFF state (black) have a nonuniform intensity profile, with significant variation in the intensity. Upon activation of the speckle scrambler (red), the profiles gain significantly in uniformity. The radially averaged intensity profiles (C) provide further evidence of the gain in uniformity. Finally, the laser beam's profile was directly imaged using a CCD beam profiler, with the speckle scrambler ON (D) and OFF (E).

The beam profile was directly imaged using a CCD beam profiler, by placing the sensor just above the objective lens, and is shown in Figure 4. The laser beam emerging from the objective was imaged without any sample loaded on the stage, to directly evaluate the effects of the speckle scrambler on the beam profile. From direct observations of the profiles with the speckle scrambler ON and OFF, shown in Figures 4(D) and (E), respectively, it is immediately evident that the introduction of the speckle scrambler effectively averages out the large variability in intensity across the beam front at intermediate length scales. Additionally, the intensity profiles taken across the centre of the images across the *x*‐ and *y*‐axes are shown in Figures 4(A) and (B), respectively, further demonstrate the increase in uniformity of the laser's beam profile. The intensity cross sections with the speckle scrambler in the ON state (red) are substantially more uniform and Gaussian compared to those in the OFF state (black). Finally, the radially averaged intensity profiles are shown in Figure 4(C). Upon activation of the speckle scrambler (red), the radially averaged intensity profile has a significant increase in uniformity compared to the profile with the speckle scrambler OFF (black) and they have reduced spatial spread. Surface plots of the projections of the laser beam are shown in Figure S1.

**Figure 5:**
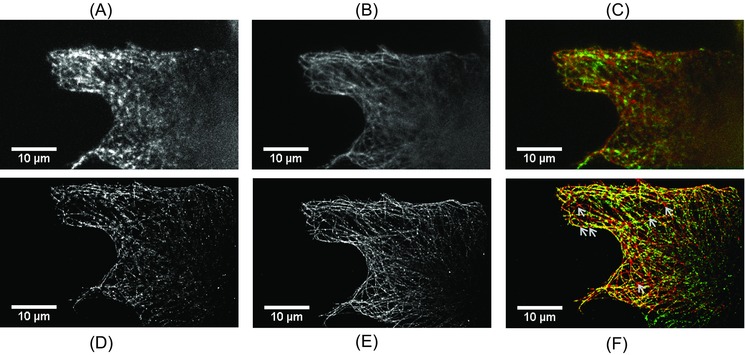
Wide‐field diffraction limited images (A)–(C) and super‐resolved reconstructions of the same FOV (D)–(F) of fixed MRC5 cells stained with DM1A α‐tubulin antibody/Alexa Fluor 647 with the speckle scrambler OFF in (A), (D) and ON in (B) and (E). In (C) and (F), the red channel in the composite images shows the image acquired with the speckle scrambler ON and the green channel with it OFF. The illumination and the resulting images are considerably more uniform with the speckle scrambler ON as the vibrating polymer membrane effectively averages over a range of speckle patterns compared to OFF. Furthermore, the reduction of the interference and speckle/fringe patterns on the imaging plane allows for the reconstruction of uniform super‐resolved images, evident from the obvious gaps in the green channel, which would appear as apparently broken microtubules in super‐resolved images.

Finally, the diffraction‐limited and super‐resolved images of fluorescently labelled microtubules in MRC5 cells are shown in Figure 5. In this case, data with the speckle scrambler ON were collected first, then it was turned OFF and the same FOV was imaged again. The 405 nm laser was used in the subsequent acquisition to compensate for photobleaching. The order that the data was collected was reversed in Figure S4 to allow for comparison and no significant changes were seen. In all reconstructions, more than 1 million localisations were used to reconstruct the super‐resolved images. The increase in the illumination uniformity is directly evident from the composite diffraction‐limited and super‐resolved images (Figs. 5C and F, respectively), where gaps in the green channel indicate nonuniform illumination due to speckle/interference patterns. Furthermore, Figure 5(D) shows a super‐resolved STORM reconstruction of the same FOV with the speckle scrambler OFF, where apparently nonuniform and broken microtubules are discerned, whereas these appear continuous when the speckle scrambler is in the ON state, as in Figure 5(E). Other representative diffraction limited and super‐resolved images are shown over more extended FOVs in the Supplementary Materials (Figs. S2–S5). Line profiles along super‐resolved microtubules with the speckle scrambler ON and OFF are shown in Figure S6. The full width at half maximum (FWHM) of Gaussian fits performed on the aforementioned profiles from adequately resolved microtubules was in the range of 55–65 nm. This number in part reflects the bulky size of the fluorescent antibody labels, ∼20 nm, since the diameter of microtubules is known to be 24 nm (Alberts *et al*., 2013).

## Discussion

Advances in fluorescence microscopy techniques have led to many discoveries in biology and medicine (Mertz, 2010; Davidson & Murphy, 2012). One of the greatest differences in modern fluorescent microscopes when compared to earlier generations is the increased use of lasers as a means of sample illumination, which offer a number of advantages compared to arc lamps, such as high spatial coherence, well defined collimation, narrow bandwidth and higher illumination intensities.

Mirrors and lenses are needed to manipulate laser beams in super‐resolution fluorescence microscopes, which cause local interference and multiple scattering effects from imperfections, dust particles and vibrations on the optical elements’ surfaces that can give rise to unwanted interference and speckle effects in the illumination plane. We used a speckle scrambler that operates by moving an electroactive polymer as a diffuser in a perpendicular plane to the light beam, effectively averaging over many speckle patterns. The speckle scrambler is placed in the intermediate focal plane of a Keplerian beam expander, where there is no extended wave front (i.e. it is held at a point of focus), and the collimated/expanded beam is then focused on the objective's back focal plane. This is observed by directly imaging the beam profile shown in Figures 4(D) and (E). Upon activation of the speckle scrambler, the radially averaged intensity profile becomes much smoother with no intensity peaks (Fig. 4C).

We evaluated the use of the speckle scrambler in a super‐resolution single molecule localisation microscope setup, both with Dextran/Alexa Fluor 647 coated glass bottom dishes and DM1A (α‐tubulin antibody) stained MRC5 cultured cells. In both cases, the use of the scrambler created more uniform illumination and eliminated the fringe/speckle patterns. This is particularly evident in Figure 2, where the histogram and intensity line profiles from the reconstructed super‐resolution images of Dextran coated dishes are shown. The distribution of the pixels’ greyscale values is more symmetrical when the speckle scrambler is introduced, whereas without it there is an exaggerated tail towards both ends of the greyscale range, as a result of uneven illumination and a subsequent decreased number of localisations in the reconstructed pattern. The intensity profiles further demonstrate the gain in uniformity, as the absence of dark fringes in the illumination plane when using the speckle scrambler result in considerably less variation between intensity maxima and minima.

Furthermore, we compared the fit results obtained using the ThunderSTORM ImageJ plugin (shown in Fig. 3). We found that the intensity in photons per localisation, Gaussian fit sigma, the localisation uncertainty, background intensity and number of localisations are comparable with and without the speckle scrambler within 1 standard deviation. The integration time used to obtain the data shown was longer than the speckle scrambler's response, thus a homogenised field of view was imaged. Using a smaller integration time, especially in the case of sCMOS cameras, which are now capable of hundreds of frames per second acquisition over considerably large field of view, would result in imaging faster than the response of the speckle scrambler. In that case, different speckle patterns will be imaged during the STORM data acquisition, which would result in needing a larger number of frames and, possibly, some postprocessing to average over them. An ultimate theoretical goal would be to reduce the size of the speckle to that of the pixel size on the detector so that it is completely invisible, but the speckle scrambler as used decreases the visibility of the fringes/speckle pattern to a negligibly low level and as such it provides a useful improvement; a step towards a rigorous quantitative elimination of all speckle effects.

Several methods have previously been implemented to overcome the problem of interference in laser illuminated fluorescent microscopes, including the use of a spinning disk in the light path and the vibration of an optical fibre to reduce the spatial and temporal coherence of the beam. These methods are known to decrease the intensity of the focused beam in the objective lens, whereas the use of relatively large inertia motors can introduce vibrations in the whole apparatus, which can potentially reduce the quality of the final images due to drift and increased noise (Mattheyses *et al*., 2010; Johnson *et al*., 2012). High‐frequency switching of lasers can reduce the longitudinal coherence length of beams and thus improve speckle problems (Dulin *et al*., 2014), but this is not possible with all lasers and can lead to instability issues such as laser pointing. The ease of analysis of the speckle scrambler images compares favourably to beam scanning methods and the instrumentation is slightly easier to align. The speckle scrambler uses an electroactive polymer, which does not depend on an external motor for its movement and thus does not introduce additional vibrations into the experiments. Furthermore, it can be directly incorporated in both open‐air and fibre coupled laser illuminated microscopes without major modifications to their optical setups. As with a number of the aforementioned methods, there is a loss of power at the objective when incorporating the speckle scrambler in the optical pathway of the microscope, due to the translating diffuser. The loss of power is on the order of 20–25%. A potential drawback is the partial loss of the beam's Gaussian profile when compared to coupling the laser beams and vibrating the optic fibre. Since modern laser‐based microscopes use lasers that span the whole visible spectrum and even span to IR, coupling all of the lasers in a single multimode fibre is often challenging and the coupling efficiency varies between the different wavelengths. We have found this is not a problem with the free‐space arrangement using the speckle scrambler if good quality achromatic lenses are used in the Keplerian beam expander.

We therefore consider the use of the polymer‐based speckle scrambler to be a cost effective and straightforward solution to eliminate nonuniformities in the illumination of super‐resolution fluorescence microscopes and it would be equally applicable to other demanding microscope‐based applications such as single‐molecule imaging using TIRF lenses. In the future we hope to combine the speckle scrambled STORM apparatus with additional modalities, that is, polarisation orientation controlled STORM, three‐dimensional imaging using a cylindrical lens (Bates *et al*., 2013) and spectrally resolved multicolour STORM (Zhang *et al*., 2015).

## Conclusions

The use of lasers in various types of modern fluorescence microscopes is steadily replacing arc lamps. However, the high spatial coherence of the light beam can give rise to unwanted interference effects, resulting in uneven sample illumination and imaging artefacts. Here, we evaluated the use of a polymer‐based speckle scrambler as a means to eliminate these interference effects in a super‐resolution fluorescence microscope (STORM). We measured both Alexa‐Fluor 647 labelled dextran monolayers and immunostained cultured cells. The speckle scrambler effectively eliminated the fringes/speckle patterns in the illumination plane at micron length scales without affecting the super‐resolving power of the microscope at distances of tens of nanometres. Beam collimation for TIRF experiments becomes slightly more challenging, but it is not a serious issue.
